# Biochemistry, proteomics, and phosphoproteomics of plant mitochondria from non-photosynthetic cells

**DOI:** 10.3389/fpls.2013.00051

**Published:** 2013-03-13

**Authors:** Jesper F. Havelund, Jay J. Thelen, Ian M. Møller

**Affiliations:** ^1^Department of Molecular Biology and Genetics, Science and Technology, Aarhus UniversitySlagelse, Denmark; ^2^Department of Biochemistry and Interdisciplinary Plant Group, University of Missouri-ColumbiaColumbia, MO, USA

**Keywords:** plant mitochondria, proteomics, mitochondrial isolation, protein phosphorylation

## Abstract

Mitochondria fulfill some basic roles in all plant cells. They supply the cell with energy in the form of ATP and reducing equivalents [NAD(P)H] and they provide the cell with intermediates for a range of biosynthetic pathways. In addition to this, mitochondria contribute to a number of specialized functions depending on the tissue and cell type, as well as environmental conditions. We will here review the biochemistry and proteomics of mitochondria from non-green cells and organs, which differ from those of photosynthetic organs in a number of respects. We will briefly cover purification of mitochondria and general biochemical properties such as oxidative phosphorylation. We will then mention a few adaptive properties in response to water stress, seed maturation and germination, and the ability to function under hypoxic conditions. The discussion will mainly focus on Arabidopsis cell cultures, etiolated germinating rice seedlings and potato tubers as model plants. It will cover the general proteome as well as the posttranslational modification protein phosphorylation. To date 64 phosphorylated mitochondrial proteins with a total of 103 phosphorylation sites have been identified.

## Introduction

All living plant cells contain mitochondria, the organelle where organic molecules are oxidized to produce energy, in the form of ATP and reducing equivalents, as well as metabolic intermediates for use in biosynthetic processes. However, mitochondria have a number of other important functions depending on the cell and tissue type, as well as environmental conditions. In theory, each of these factors affects the properties of the mitochondria and the composition of its proteome. While this has been demonstrated at the biophysical level (Douce and Neuburger, 1989) and for some enzymes and complexes, we are entering an era where comprehensive protein profiling is possible to quantitatively assess mitochondrial remodeling in response to genetic and environmental cues. In anticipation and preparation for this époque we review critical aspects of the field including: (1) methods for isolation of ultra-pure mitochondria (for proteome interrogation); (2) properties of mitochondria (to characterize function); and (3) the current status of mitochondrial proteome investigations. Due to space constraints, we focus this review on mitochondria from non-photosynthetic tissues, which can be considered the “reference state” for comparative studies. These mitochondria perform the same basic functions and would therefore be expected to contain many of the same proteins as mitochondria from photosynthetic tissues.

## Purification of mitochondria from non-green tissues

As is the case for all studies of organellar proteomics, a mitochondrial isolation procedure must remove all contaminants and give a clean preparation containing only mitochondria. Biochemical studies of mitochondrial metabolism often require several mg protein. Therefore, access to large amounts of starting material has been an important factor when selecting the species and tissue to study.

During the 1950–1970's, non-photosynthetic tissues such as etiolated seedlings or storage tissues were usually employed for the isolation of mitochondria by differential centrifugation probably because a major, visible contaminant—thylakoid membranes—was absent. Actually, crude mitochondria from storage tissues are generally contaminated by amyloplast membranes (Neuburger et al., 1982), but they do not interfere with measurements of oxygen consumption, a standard method in the study of mitochondrial metabolism. When rate-zonal, density gradient purification of plant mitochondria was introduced, it was initially applied to potato tubers. Step sucrose gradients were first introduced (Douce et al., 1972) and later Percoll gradients (Neuburger et al., 1982; Struglics et al., 1993; Considine et al., 2003), which had the great advantage over sucrose gradients that the mitochondria were not exposed to large changes in osmolarity. Not only does Percoll gradient centrifugation remove major contaminants like plastids and peroxisomes, it also removes mitochondria with damaged outer and/or inner membranes (Neuburger et al., 1982; Struglics et al., 1993).

Using Percoll gradient centrifugation, purified mitochondria have now been isolated from a range of non-green tissues from a number of species (Moreau and Romani, 1982; Liden and Møller, 1988; Fredlund et al., 1991; Lind et al., 1991; Millar et al., 2001; Bardel et al., 2002; Robson and Vanlerberghe, 2002; Qin et al., 2009; Lee et al., 2011). This has usually given a very significant improvement in mitochondrial purity and intactness and a concomitant increase in rates of respiration, which indicates that the crude mitochondria previously used had sometimes contained less than 50% undamaged mitochondria, on a protein basis (e.g., Moreau and Romani, 1982). In the case of purified potato tuber mitochondria, contamination by plastid envelope and peroxisomes could be calculated to be <0.5% on a protein basis (Neuburger et al., 1982; Struglics et al., 1993). Percoll-purified mitochondria from Arabidopsis cell cultures, roots and shoots can be further purified by free-flow electrophoresis (Eubel et al., 2007; Lee et al., 2011). For cell culture mitochondria, this method decreases the contamination by peroxisomal and plastidic proteins by an estimated 5- to 10-fold (Eubel et al., 2007).

## Biochemical properties of mitochondria from non-green tissues

### Basic properties

Mitochondria are semi-autonomous, membrane-bound organelles. Consistent with their endosymbiotic origin, plant mitochondria contain small circular genomes. Varying with species, 30–40 proteins are encoded in the mitochondrial DNA, transcribed and translated in the matrix on bacterial-like ribosomes (Kubo and Newton, 2008). The remaining proteins making up the mitochondrial proteome are imported from the cytosol mainly through two large protein complexes, TIM and TOM (Translocator Inner/Outer Membrane) (Lister et al., 2005).

Isolated mitochondria from non-green cells and tissues oxidize a variety of substrates, including most of the tricarboxylic acid (TCA) cycle intermediates, with good coupling indicating that the TCA cycle is fully functional and that the four electron transport chain (ETC) complexes are present together with ATP synthase (Douce and Neuburger, 1989). In addition, the ETC of mitochondria from non-green tissues contain up to four alternative NAD(P)H dehydrogenases (DH) (Rasmusson et al., 2004), the alternative oxidase (Vanlerberghe and McIntosh, 1997), and has the uncoupling protein associated with it (Vercesi et al., 1995; Zhu et al., 2011). A range of DHs in the matrix feed electrons into the ETC at ubiquinone (UQ)—proline DH, lactate DH, and DHs involved in branched amino acid degradation via electron-transfer flavoprotein and electron-transfer flavoprotein: quinone oxidoreductase. On the outer surface of the inner membrane glycerol-3-phosphate DH also feeds electrons into the UQ pool, while L-galactono-1,4-lactone DH, the last enzyme in ascorbate biosynthesis, feed electrons into cytochrome c. The presence and amount of these enzymes in the mitochondria depends on species, tissue and environmental conditions (reviewed by Rasmusson et al., 2008; Rasmusson and Møller, 2011). To interact with other subcellular compartments within the cell, the inner mitochondrial membrane contains a number of transport proteins for the exchange of metabolites, coenzymes, etc. (Laloi, 1999; Palmieri et al., 2009).

Isolated mitochondria from green tissues do not differ much in the basic properties of the ETC and the TCA cycle. However, “green” mitochondria differ in the balance between the different substrates used. This is especially true for glycine, the product of photorespiration, which is oxidized at high rates by “green” mitochondria, but also their amino acid metabolism differs (e.g., Lee et al., 2008).

### Responses to hypoxia, drought, and desiccation

Plants can be exposed to hypoxic conditions as a result of flooding (Greenway et al., 2006). However, even under normoxic external conditions, the central parts of dense, metabolically active and/or bulky non-photosynthetic tissues can experience hypoxic conditions because the diffusion of oxygen into the tissue cannot keep up with the rate of removal by respiration (Geigenberger, 2003). This leads to metabolic changes such as the induction of ROS-degrading enzymes, presumably to prevent post-anoxic injury (Geigenberger, 2003), and higher activity of the enigmatic formate dehydrogenase (FDH) (Bykova et al., 2003b), one of the most abundant proteins in potato tuber mitochondria (Colas Des Francs-Small et al., 1993). The function of FDH may be to remove formate, but the source of formate is unknown and the reason the enzyme is so abundant is obscure (Igamberdiev, 1999; Ambard-Bretteville et al., 2003).

Drought presents the plant with quite a different problem and production of compatible solutes, such as proline, is one strategy to ameliorate the effects of a low water potential. As mitochondria are involved in proline turnover it is likely that homeostatic balance of this osmolyte requires the coordinated action of multiple organelles (Atkin and Macherel, 2009).

Mitochondria in maturing and germinating seeds experience quite unique conditions. The water content during the late-maturation phase is very low and a late-embryogenesis-abundant (LEA) protein is induced to protect mitochondrial membranes (Macherel et al., 2007; Tolleter et al., 2007). At the same time, the mitochondria presumably have to cope with hypoxic conditions caused by their own activity (Borisjuk and Rolletschek, 2009).

## Proteomics of mitochondria from non-green tissues

The most comprehensive proteomic studies in non-photosynthetic tissues have been performed on mitochondria from Arabidopsis suspension cells, a de-differentiated cell type that, when grown in darkness with sugar, is non-photosynthetic. For this reason and space considerations, this discussion of proteomics and post-translational modifications will focus mainly on Arabidopsis suspension cells and etiolated rice seedlings.

Bioinformatic analysis of the Arabidopsis genome, using primarily prediction algorithms, has estimated that mitochondria may contain as many as 2000–3000 different proteins (Millar et al., 2005, 2006; Cui et al., 2011). It is surprising then that nearly 20 years into the proteomics era no more than 500–600 different mitochondrial isolation proteins have been experimentally confirmed from any plant mitochondrial source! This is not due to limitations in technology, as it is now routine to not only identify, but quantify, at least 1900 proteins from a single biological sample using standard tandem mass spectrometry (e.g., Balbuena et al., 2012). We therefore expect the gap between the number of experimental and predicted mitochondrial proteins to shrink in the coming years.

The most comprehensive proteomic characterization of purified mitochondria from non-photosynthetic cells has been from Arabidopsis suspension cells (Heazlewood et al., 2004). A total of 390 unique proteins were qualitatively identified by shotgun, reversed-phase (RP) LC-MS/MS analysis of 15–20 μg of tryptic peptides. Diverse groups of proteins were identified including proteins involved in energy and metabolism, DNA replication, transcription, translation, protein complex assembly, and signaling, as well as approximately 70 proteins of unknown function. Interestingly, various glycolytic enzymes were detected in mitochondrial preparations from Arabidopsis suspension cells (Heazlewood et al., 2004). Subsequent studies verified that these enzymes are indeed associated with mitochondria through scaffold proteins, such as the voltage-dependent anion channel (VDAC) located in the outer membrane (Graham et al., 2007). Additionally, *in vivo* association of cytosolic glycolytic enzymes with mitochondria is a dynamic process allowing for respiration to be supported in a dedicated manner consistent with substrate channeling (Graham et al., 2007). Although many of the individual subunits of the five respiratory complexes were identified by Heazlewood et al. (2004), follow-up proteomic analysis of these complexes was necessary to reveal the full protein complements (Meyer et al., 2008; Klodmann and Braun, 2011). In a study focusing on mitochondrial protein complexes, (Klodmann et al., 2011) identified 471 non-redundant proteins belonging to at least 35 different protein complexes. Subsequent proteomic analysis of purified outer membranes also expanded the compendium of proteins mapped to these mitochondrial subcompartment especially integral membrane proteins (Duncan et al., 2011; Tan et al., 2012).

Besides Arabidopsis roots and suspension cells, mitochondria from etiolated rice shoots have also been characterized at the proteome level (Bardel et al., 2002; Huang et al., 2009). Using both 2D gel electrophoresis and RP-LC-MS/MS analyses a total of 322 non-redundant proteins were identified in a non-quantitative manner. Comparison with Arabidopsis cell culture mitochondria (Heazlewood et al., 2004) revealed a similar cohort of proteins, although 20% of the rice mitochondrial proteins did not produce orthologous matches to the Arabidopsis mitochondrial proteome (Huang et al., 2009). And like the Arabidopsis experimental proteome, approximately 60% of rice mitochondrial proteins were predicted to be targeted to this organelle using various organelle prediction algorithms. Despite the wealth of shotgun and targeted mitochondrial proteomic studies it is obvious that a comprehensive compendium of experimentally-verified plant mitochondrial proteins is currently unavailable. This is apparent when one not only considers the gap between predicted and experimentally identified proteins, but also from perusing the current catalogue of approximately 500 mapped proteins. Missing from this list are many essential plant mitochondrial activities including regulatory proteins, transcription factors, metabolite translocators, and the wealth of tRNA synthases and pentratricopeptide repeat proteins. For example, the two regulatory enzymes of the pyruvate dehydrogenase complex (PDC)—the PDC kinase and phospho-PDC phosphatase, which are activities in mitochondria from both green and non-green tissues (Thelen et al., 1998a,b)—are also undetected in global plant mitochondrial proteomic studies.

While the early stages of non-photosynthetic mitochondrial proteome characterization dealt with protein cataloguing, more recent research has shifted toward comparative analyses to discover dynamic changes in mitochondrial protein expression. Proteomic comparisons of mitochondria from non-photosynthetic Arabidopsis suspension cells and developing photosynthetic shoots (Lee et al., 2008) as well as developing Arabidopsis roots and photosynthetic shoots (Lee et al., 2011) collectively revealed differences in TCA cycle and photorespiratory enzymes. In both instances, most of the component enzymes of the glycine decarboxylase complex, except for the lipoamide dehydrogenase (E3) subunit, which is shared with the PDC, were highly upregulated in green shoot mitochondria compared to the two non-photosynthetic counterparts. Additionally, FDH was highly induced in mitochondria from green shoots compared to roots and suspension cells. In contrast, three subunits of the PDC complex including E3 were more prominently expressed in root and suspension cell mitochondria. These and other changes to TCA and amino acid metabolism between mitochondria from green and non-green sources confirmed previous observations (e.g., upregulation of GDC in photorespiring tissues) but also suggested previously unknown differences in both carbon import/export and oxidative metabolism for this organelle as a direct result of photosynthetic capacity.

Additional comparative proteomic studies have been performed with mitochondria from etiolated rice shoots, analyzing the effect of a hypoxic/anoxic environment (Millar et al., 2004; Howell et al., 2007). In the absence of oxygen, mitochondrial respiration was impaired due to lower activity and expression of respiratory complexes cytochrome bc_1_ and cytochrome c oxidase (Millar et al., 2004). Additionally, the E1β subunit of the PDC and a putative succinyl-CoA ligase (GDP-forming) β-chain as well as mitochondrial processing peptidase α- and β-chain subunits (a component of the cytochrome bc_1_ complex) were reduced in expression (Howell et al., 2007). In contrast, a TIM subunit was highly induced under anaerobic conditions. It was concluded that under anoxic conditions a direct link between respiratory capacity and protein import could be established at the cytochrome bc_1_ complex of the ETC. While total proteome coverage was not attained in either of these studies, the results illustrate the potential for comparative proteomics to elucidate the dynamic properties of mitochondria.

## Protein phosphorylation in plant mitochondria

Several hundred posttranslational modifications (PTMs)—the covalent addition of a chemical group to amino acids in proteins–are known. They often lead to alterations in properties and function of the proteins and are therefore involved in the regulation of large variety of important biological processes (Wold, 1981; Mann and Jensen, 2003). The most well-studied PTM is phosphorylation, which is catalyzed by protein kinases (PKAs) while the dephosphorylation is catalyzed by protein phosphatases. This modification primarily targets the hydroxyl groups of Ser, Thr, and Tyr residues and has long been known to be a key player in signaling. Both protein phosphorylation and other PTMs such as acetylation are important in mammalian mitochondria (Guan and Xiong, 2011; Koc and Koc, 2012), but in plant mitochondria only protein phosphorylation has been studied in any detail.

Traditionally, the methods for detecting phosphoproteins are based on incorporation of radioactive phosphate (^32^P). This is either done by *in vitro* phosphorylation reactions using protein extracts or isolated organelles or by a more physiologically relevant *in vivo* approach using cells. The phosphorylated proteins are then typically separated by gel electrophoresis and detected by phosphoimaging or autoradiography (Thelen et al., 2000; Bykova et al., 2003a; Berwick and Tavare, 2004). A fluorescent phosphosensor dye called Pro-Q Diamond has also been developed (Schulenberg et al., 2003; Ito et al., 2009). The phosphoproteins can be cut out of the gel and identified by LC-MS/MS (Pappin et al., 1993; Heazlewood et al., 2004; Ito et al., 2009). However, more recently shotgun methods of identifying phosphopeptides and phosphoproteins first uses a phosphopeptide enrichment method such as immobilized metal affinity chromatography (IMAC) (Posewitz and Tempst, 1999; Stensballe et al., 2001) or titanium oxide chromatography (MOC) followed by LC-MS/MS. In this way thousands of phosphorylation sites can be identified without the use of gel electrophoresis (e.g., Engholm-Keller et al., 2012; Meyer et al., 2012).

To date, 64 plant mitochondrial proteins have been reported to be phosphorylated on 103 phosphosites (Table 1). The distribution of these on Ser, Thr, and Tyr residues is 81, 14, and 5%, respectively, or close to the distribution in all the known 22,995 eukaryotic phosphoproteins with 100,281 phosphosites (Rao and Møller, 2012) in spite of the fact that plants do not contain canonical Tyr kinases (Yao et al., 2012).

**Table 1:** **Phosphoproteins and phosphosites in plant mitochondria**.

**No**	**Protein**	**Plant species**	**Phosphorylation site**	**References**
**ENERGY**				
1	Pyruvate dehydrogenase (EC 1.2.4.1), E1α-subunit	Potato	Ser^298^: YHGH**(S)**MSDP	1, 3, 4^a^
		Arabidopsis	Ser^292^: YHGH**(S)**MSDP	5^a^, 10^a^, 9^a^, 11^a^
		Pea		14
		Rice	Ser^110^: AITR**(S)**D(S)II	12^a^
			Ser^112^: TR(S)D**(S)**IITA	
			Ser^293^: YHGH**(S)**M(S)D	
			Ser^295^: GH(S)M**(S)**DPGS	
2	Aconitate hydratase (EC 4.2.1.3)	Potato		3
3	NAD-isocitrate dehydrogenase (EC 1.1.1.41)	Potato		3
4	Succinyl-CoA-ligase (EC 6.2.1.5), α-subunit	Potato		3
		Arabidopsis	Ser^304^: GAIV**(S)**GGKG	9^a^, 12^a^
		Rice	Ser^289^: GAIV**(S)**GGKG	12^a^
			Ser^312^: TVVE**(S)**PAKI	
5	Succinyl-CoA-ligase (EC 6.2.1.5), β-subunit	Potato		3
6	Succinate dehydrogenase (EC 1.3.5.1), flavoprotein	Potato		3
		Arabidopsis		5
		Rice	Ser^161^: FGGQ**(S)**LDFG	12^a^
7	Succinate dehydrogenase (EC 1.3.5.1), subunit 5	Arabidopsis		5
8	Malate dehydrogenase (EC 1.1.1.37)	Potato		3
		Arabidopsis	Ser^23^: RRSF**(S)(**S)GSV	9^a^
			Ser^24^: RSF(S)**(S)**GSVP	
			Ser^314^: LGPL**(S)**DFEK	
		Rice	Ser^313^: LGQL**(S)**DFEK	12^a^
9	NAD-malic enzyme (EC 1.1.1.38), 62 kDa isoform	Potato		3
10	NAD-malic enzyme (EC 1.1.1.38), 59 kDa isoform	Potato		3
11	Protein NADH-ubiquinone oxidoreductase (EC 1.6.5.3), 39 kDa subunit	Rice	Ser^397^: PAFG**(S)**(T)V(S)E	12^a^
			Thr^398^: AFG(S**)(T)**V(S)EK	
			Ser^400^: G(S)(T)V**(S)**EKIR	
12	Complex III (EC 1.10.2.2), β-mitochondria processing peptidase subunit	Potato		3
		Rice	Ser^485^: SWFR**(S)**HTY(S)	
			Ser^489^: (S)HTY**(S**)DDEF	12^a^
13	Protein mitochondrial-processing peptidase (EC 3.4.24.64), α-subunit	Rice	Ser^234^: HRLD**(S)**(S)ILE	12^a^
			Ser^235^: RLD(S)**(S)**ILEE	
14	F_1_ ATPase (EC 3.6.1.3), δ-subunit	Potato		2
15	ATP synthase (EC 3.6.1.3), α-subunit	Potato		3
16	ATP synthase (EC 3.6.1.3), β-subunit	Rice	Ser^355^: GRIP**(S)**AVGY	12^a^
			Thr^362^: GYQP**(T)**LATD	
			Ser^418^: SRQI**(S)**ELGI	
17	F_0_- ATPase (EC 3.6.1.3), b/C/D-subunit	Potato		2
		Rice	Ser^157^: KAVD**(S)**LVPI	12^a^
		Arabidopsis	Ser^77^: VENK**(S)**QGSE	9^a^
			Ser^80^: K(S)QG**(S)**EVLL	
			Ser^386^: EKSE**(S)**LEEI	
		Arabidopsis	Ser^248^: QRGV**(T)**LGDP	10^a^
18	Formate DH (EC 1.2.1.2)	Potato	Thr^76^: QYIV**(T)**PDKE	3, 4^a^
			Thr^333^: NQAM**(T)**PHIS	
		Arabidopsis		5
19	Glutamate DH 1 (EC 1.4.1.2)	Arabidopsis		5
20	Glutamate DH 2 (EC 1.4.1.2)	Arabidopsis		5
21	Mitochondrial lipoamide dehydrogenase 1 (EC 1.8.1.4)	Arabidopsis	Ser^319^: TPFT**(S)**GLDL	9^a^, 12^a^
**METABOLISM**				
22	Protein cysteine synthase (EC 2.5.1.47)	Rice	Ser^130^: PAGY**(S)**LDKQ	12^a^
**PROTEIN FATE**				
23	Heat shock protein 90	Potato		3
		Arabidopsis	Ser^219^: EKEI**(S)**DDEE	5^a^, 9^a^, 10^a^, 11^a^
24	Heat shock protein 70	Potato		3
		Bean		6
		Rice	Ser^419^: GLSE**(S)**DIEK	12^a^
			Ser^526^: GSSS**(S)**(S)GGD	
			Ser^527^: SSS(S)**(S)**GGDQ	
	Heat shock protein 70-1	Arabidopsis	Ser^398^: QEIV**(S)**EIFGK	9^a^
			Ser^404^: IFGK**(S)**PCKG	
			Ser^552^: SGGL**(S)**DDEI	
	Heat shock protein 70-2	Arabidopsis	Ser^409^: IFGK**(S)**PSKG	12^a^
25	Chaperonin 60	Potato		3
	60 kDa chaperonin 7	Rice	Ser^137^: QSYG**(S)**PKV	12^a^
			Thr^522^: DKLP**(T)**ANFD	
26	Mitochondrial small heat shock protein	Potato		3
	Mitochondrial small heat shock protein, 22 kDa	Maize		7
		Rice	Ser^58^: GRLL**(S)**LMDD	12^a^
			Ser^101^: ERDE**(S)**DDDS	
27	26.2 kDa heat shock protein	Rice	Tyr^71^: PLRR**(Y)**DVVD	12^a^
			Ser^77^: VVDE**(S**)G(T)D(S)	
			Thr^79^: DE(S)G**(T)**D(S)GD	
			Ser^81^: (S)G(T)D**(S)**GDE(Y)	
			Tyr^85^: (S)GDE**(Y)**DA(T)D	
			Thr^88^: E(Y)DA**(T)**DDGR	
28	GRPE co-chaperone	Arabidopsis		5
29	Protein GrpE 1	Rice	Ser^81^: SPEL**(S)**DKEE	12^a^
30	Lon1 protease (EC 3.4.21.53)	Arabidopsis	Ser^74^: KAVE**(S)**DSEV	5^a^, 9^a^
**TRANSCRIPTION**				
31	Mitochondrial transcription termination factor family protein [At1g62010]	Arabidopsis	Ser^53^: FKSS**(S)**FLDS	12^a^
32	Transcription termination factor domain-containing protein [AT2G44020]	Arabidopsis	Thr^488^: GEIV**(T)**DEEE	12^a^
			Ser^495^: EEDE**(S)**DDEV	
33	Mitochondrial transcription termination factor family protein [At2g21710]	Arabidopsis	Tyr^501^: DEVL**(Y)**RRTL	9^a^
			Tyr^168^: FGIT**(Y)**A(T)NV	
			Thr^170^: IT(Y)A**(T)**NVTD	
**RNA PROCESSING**				
34	Putative mitochondrial RNA helicase (EC 3.6.4.13), helicase 2	Arabidopsis	Ser^603^: GGRS**(S)**FGGF	12^a^
35	Protein ATP-dependent RNA helicase (EC 3.6.4.13), SUV3	Rice	Ser^693^: VEQA**(S)**DDNA	12^a^
**PROTEIN SYNTHESIS**				
36	Glycine rich RNA-binding protein 3	Arabidopsis		5
37	Ribosomal protein S24/S35	Arabidopsis	Thr^117^: KHAE**(T)**DDEL	5^a^, 9^a^, 10^a^, 12^a^
38	Protein glycyl-tRNA synthetase 2 (EC 6.1.1.14)	Rice	Ser^721^: QKKL**(S)**EFRD	12^a^
39	Protein glycyl-tRNA synthetase 1 (EC 6.1.1.14)	Rice	Ser^671^: EEEA**(S)**E(T)..	12^a^
			Thr^673^: EA(S)E**(T)** ….	
40	Protein rf1 protein	Rice	Ser^361^: DLMV**(S)**RGCY	12^a^
**DEFENSE, STRESS, DETOXIFICATION**				
41	Mn superoxide dismutase (1.15.1.1)	Potato		3
**COMMUNICATION/SIGNALING**				
42	MAM protein	Arabidopsis	Ser^94^: DNID**(S)**DEEM	5
43	MAM 33	Arabidopsis		5
44	Protein calcium-binding mitochondrial protein Anon-60Da	Rice	Ser^609^: ASAS**(S)**VSKE	12^a^
45	Protein protein-tyrosine phosphatase 1 (EC 3.1.3.48)	Rice	Ser^269^: CLIP**(S)**LK..	12^a^
**TRANSPORT**				
46	Mitochondrial glycoprotein family protein [AT4G32605.1]	Arabidopsis	Ser^76^: SLEG**(S)(**T)GAV	9^a^
			Thr^77^: LEG(S)**(T)**GAVL	
47	Mitochondrial glycoprotein family protein [AT2G39795]	Arabidopsis	Ser^84^: DNID**(S)**DEEM	5^a^, 12^a^
48	Protein mitochondrial glycoprotein, expressed [BAD33248.1]	Rice	Ser^88^: RVID**(S)**EIEC	12^a^
			Ser^96^: CIVQ**(S)**EEGA	
49	Protein mitochondrial glycoprotein, expressed [A2ZP51.1]	Rice	Ser^81^: FAEE**(S)**DDHD	12^a^
50	Protein mitochondrial glycoprotein, expressed [BAF25817.1]	Rice	Ser^115^: RRVE**(S)**LERG	12^a^
51	Mitochondrial substrate carrier family protein	Arabidopsis	Ser^64^: RGNN**(S)**FSTQ	12^a^
52	Mitochondrial import inner membrane translocase, subunit TIM17/TIM22/TIM23	Arabidopsis	Ser^15^: DPSS**(S)**PPPI	12^a^
53	Mitochondrial import inner membrane translocase, subunit TIM23	Rice	Ser^8^: PRLF**(S)**(S)G(S)G	12^a^
			Ser^9^: RLF(S)**(S)**G(S)GS	
			Ser^11^: F(S)(S)G**(S)**GTRD	
		Arabidopsis	Ser^126^: AGIE**(S)**GVVA	13^a^
			Ser^140^: DVWT**(S)**VVAG	
54	Translocase of outer membrane, subunit TOM20-4	Arabidopsis	Ser^143^: AGPS**(S)**NSAK	9^a^
55	AMP deaminase (EC 3.5.4.6)	Arabidopsis	Tyr^70^: VNDQ**(Y)**GR(S)P	12^a^
			Ser^73^: QYGR**(S)**PASL	9^a^, 12^a^
			Ser^76^: R(S)PA**(S)**LPDA	12^a^
			Ser^140^: VRPI**(S)**PKSP	9^a^, 10^a^, 12^a^
			Ser^203^: IRSH**(S)**VSGD	5^a^, 12^a^
			Ser^279^: ISDP**(S)**(T)PKP	12^a^
			Thr^280^: SDP(S)**(T)**PKPN	12^a^
		Rice	Ser^94^: KPII**(S)**PAST	12^a^
			Ser^126^: TIED**(S)**DDDD	
			Ser^178^: IRSQ**(S)**ATG(S)	
			Ser^182^: (S)ATG**(S)**LHGA	
			Thr^256^: TDPS**(T)**PKPN	
56	Mitochondrial outer membrane protein porin 5	Rice	Ser^96^: KLVT**(S)**VKLP	12^a^
57	Mitochondrial outer membrane protein porin 6	Rice	Ser^95^: KTSF**(S)**FRVP	12^a^
58	Protein mitochondrial 2-oxoglutarate/malate carrier protein	Rice	Ser^97^: ARLG**(S)**FRVL	12^a^
59	Protein mitochondrial carrier protein	Rice	Ser^419^: ARAN**(S)**IEVS	12^a^
60	Mitochondrial carrier protein	Arabidopsis	Ser^155^: AAPE**(S)**PSSN	5^a^
61	TraB family protein	Arabidopsis	Ser^64^: DSVF**(S)**GGDG	12^a^
**STRUCTURE ORGANIZATION**				
62	Protein inner membrane protein OXA1L	Rice	Ser^422^: SQRF**(S)**DLEN	12^a^
**MISCELLANEOUS**				
63	Stomatin-like protein	Arabidopsis	Thr^362^: ALDE**(T)**DLEE	5^a^
64	Prohibitin	Rice		8

This list has been compiled by first manually going through the phosphoproteomic literature on non-photosynthetic plant organisms published before 2008. Secondly, the identified proteins were used as search input in the Plant Protein Phosphorylation Database and Yao et al. (2012)—http://p3db.org/- resulting new phosphosites were included in the list. Lastly, the mitochondrial proteins from Duncan et al. (2011) were searched against P^3^DB and the hits included. The blank spaces in the column labeled Phosphorylation site indicate that the phosphoprotein, but not the specific site of phosphorylation was identified.

All phosphorylation sites are highlighted with parentheses. The specific phosphorylation site is highlighted with bold.

a This reference includes phosphosite information.

1, (Sommarin et al., 1990); 2, (Struglics et al., 1998); 3, (Bykova et al., 2003a); 4, (Bykova et al., 2003b); 5, (Ito et al., 2009); 6, (Vidal et al., 1993); 7, (Lund et al., 2001); 8, (Takahashi et al., 2003); 9, (Sugiyama et al., 2008); 10, (Li et al., 2009); 11, (de la Fuente van Bentem et al., 2008); 12, (Nakagami et al., 2010); 13, (Meyer et al., 2012); 14, (Budde and Randall, 1990).

The largest group of phosphorylated proteins is energy and transport (Table 1), a trend also observed in yeast (Reinders et al., 2007). Phosphorylation sites have been identified on most of the TCA cycle enzymes, and on complexes III–V, whereas no phosphoproteins have been found in Complexes I and II. It seems likely that oxidative phosphorylation is regulated by reversible protein phosphorylation. Other major processes that may be regulated by phosphorylation are transcription, translation, protein folding, and metabolite transport across the inner mitochondrial membrane. In mammalian mitochondria, translation is regulated by protein phosphorylation (Koc and Koc, 2012).

PDC, which produces the acetyl-CoA entering the TCA cycle, is the only enzyme in plant mitochondria where the detailed regulation by reversible phosphorylation/dephosphorylation has been elucidated (Rubin and Randall, 1977; Budde and Randall, 1990). When PDC products accumulate, they activate the PDC kinase, which phosphorylates and inactivates the PDH component of PDC (Tovar-Mendez et al., 2003). In contrast, PDC substrates inhibit the PDC kinase and stimulate the phospho-PDC phosphatase to dephosphorylate PDH and give a higher activity (Randall et al., 1981; Schuller and Randall, 1990; Moore et al., 1993). FDH phosphorylation appears to be regulated in a similar way by some of the same metabolites, but the effect on FDH activity is still unknown (Bykova et al., 2003b).

In yeast, phosphorylation of TOM22, Mim1, and TOM79 by casein kinase 2 (CK2) and PKA have been shown to regulate the function of TOM. CK2 promotes biogenesis of specific TOM complexes and CK2 inhibits specific TOM receptor activities and thereby the import of mitochondrial metabolite carriers. Together these phosphorylations regulate protein homeostasis (Schmidt et al., 2011). Another study has shown that phosphorylation of subunit *g* of the yeast ATP synthase subunit F_*o*_ inhibits dimerization of the ATP synthase and is thus involved in the regulation of the bioenergetic state of mitochondria (Reinders et al., 2007). Phosphorylated TOM and ATP synthase F_*o*_ subunits have also been found in plant mitochondria from non-photosynthetic cells (Table 1) and the function of these phosphorylations might be similar to that in yeast. Finally, the interaction of chaperone HSP90 with co-chaperones is regulated by phosphorylation in mammalian mitochondria (Mollapour et al., 2011; Johnson, 2012). HSP90 is one of the identified phosphoproteins in plant mitochondria (Table 1), but the site of phosphorylation so far identified is different from that of mammalian HSP90 (Mollapour et al., 2011), so the regulatory mechanism may not be the same.

Considering that plant mitochondria contain so many phosphoproteins, we might expect to find a substantial number of PKAs in plant mitochondria as well as a somewhat smaller number of protein phosphatases (Juszczuk et al., 2007). However, even in the most extensive proteomic studies published to date only ten protein kinases have been identified and no protein phosphatases (Heazlewood et al., 2004; Duncan et al., 2011; Taylor et al., 2011). This may indicate that each enzyme is present in very few copies so that they are below the technical detection limit. Alternatively there are actually relatively few kinases and phosphatases each with a relatively broad specificity. Future in depth proteomic studies will no doubt give us more information about such regulatory pathways.

### Conflict of interest statement

The authors declare that the research was conducted in the absence of any commercial or financial relationships that could be construed as a potential conflict of interest.
